# Colony size as a predictor of breeding behaviour in a common waterbird

**DOI:** 10.1371/journal.pone.0241602

**Published:** 2020-11-02

**Authors:** Piotr Minias, Kamila Gach, Radosław Włodarczyk, Maciej Bartos, Joanna Drzewińska-Chańko, Miłosz Rembowski, Dariusz Jakubas, Tomasz Janiszewski

**Affiliations:** 1 Department of Biodiversity Studies and Bioeducation, Faculty of Biology and Environmental Protection, University of Łódź, Łódź, Poland; 2 Department of Vertebrate Ecology and Zoology, Faculty of Biology, University of Gdańsk, Gdańsk, Poland; MARE – Marine and Environmental Sciences Centre, PORTUGAL

## Abstract

The choice of colony size may have profound consequences for individual fitness in colonially breeding birds, but at the same time it may require certain behavioural adaptations. Here, we aimed to examine behavioural divergence of common terns *Sterna hirundo* nesting in colonies of different size. For this purpose, we promoted establishment of small (<35 pairs) and large (>100 pairs) tern colonies under uniform ecological and environmental conditions by providing attractive patches of nesting substrate (floating rafts) at a single site. We combined video recording and GPS-tracking to assess communal and individual defence initiation rate, intra-specific aggression rate, and foraging flight characteristics. We found that birds from larger colonies more frequently engaged in communal defence and they performed longer foraging flights, while terns from smaller colonies more frequently showed individual defence behaviours. Also, intra-specific aggression rate was higher in smaller colonies, but this effect was primarily attributed to a higher proportion of edge breeding pairs, which were more aggressive. Our results suggest that various colony sizes may be associated with different behavioural syndromes, which comprise of diverse personality traits, such as social responsiveness, social tolerance, or propensity for aggression. It remains to be tested whether these behavioural differences reflect processes of phenotypic sorting among colonies of different size or whether they are a result of behavioural plasticity under different social contexts.

## Introduction

Extraordinary variation in the size of avian reproductive groups has long perplexed ecologists and evolutionary biologists [1]. Avian colonies vary greatly in size not only between- but also within-species, spanning several orders of magnitude in many taxa [2]. Some species are facultatively colonial, as they can breed solitarily or in aggregations depending on habitat and geographical location, while others can change from solitary to colonial breeding in consecutive years [3, 4]. Thus, it is generally agreed that colony size is an evolutionarily labile trait with weak phylogenetic signal, although it shows some species-specificity [5, 6]. At the same time, colony size is perceived as a dynamic, ecologically-driven trait, being shaped by a complex network of within- and between-species interactions (e.g. conspecific attraction, nest site competition, predation, parasitism) and by the environment (e.g. climate, resource availability) [1, 6].

While fitness consequences of colony size variation received a non-negligible scientific attention over the last decades [e.g. 7–10], much less empirical information is available on how the level of sociality affects reproductive behaviour of birds. Colonies can act as information centres, where information on unpredictable food resources can be transferred between individuals, thus affecting their foraging behaviour [11, 12], but one of the most common behavioural adaptations associated with colonial breeding is antipredator defence via communal mobbing [13]. It is expected that general efficiency of communal defence should correlate with group size, as the likelihood of an early detection of a diurnal predator and chances of its successful deterrence increase with the number of individuals involved in defence activities [14, 15; but see 16]. At the same time, larger group size may be associated with reduced individual investment in defence behaviour, because smaller proportion of birds can mob simultaneously under predatory disturbance, as well as predatory disturbances can last shorter (mobbing is more effective) or be less frequent (predators may avoid larger aggregations). Thus, breeding in large social groups may reduce the costs of nest defence, such as the time and energy invested and risk of injury or death during mobbing [15, 17]. Consequently, per capita cost/benefit ratio of antipredatory defence is expected to depend strictly on group size and this association may be reflected in other reproductive behaviours of birds. For example, lower per capita time investment in defence activities (including vigilance and mobbing) may allow birds to spend more time on other vital activities, such as foraging, resulting in longer and more distant flights for food. On the other hand, it may be associated with some trade-offs, as larger breeding colonies may be characterized by higher intra-specific competition over food, partners and nesting sites compared to the smaller ones [18, 19]. Also, it has been shown that large reproductive aggregations may require specific hormonal adjustments, e.g. higher testosterone levels, which is likely to increase the rate of aggression necessary to occupy and maintain attractive nest sites or mates under elevated intra-specific competition [20].

Associations between mobbing and group size have been reported for several avian species, but they were primarily based on direct behavioural comparisons between different colonies or reproductive groups [e.g. 15, 21–23]. Inferences drawn from such comparisons suffer from an inevitable limitation, as different locations may substantially vary in the composition and density of predator communities, which can have a major effect on behaviour of their potential prey [24]. Patterns emerging from experimental studies performed across colonies may also be affected by between-site variation in habitat structure or phenotypic (e.g. physiological and hormonal parameters) and genetic characteristics of nesting birds [25]. This is especially likely in species where individuals are non-randomly distributed among the colonies with respect to their quality, consistently with the assumptions of the despotic distribution (dominants secure high quality territories and force inferior competitors into unfavourable habitats [26]), which has been reported for several colonial avian species [27–29].

The aim of this study was to examine behavioural divergence of common terns *Sterna hirundo* nesting in colonies of different size under uniform environmental conditions. For this purpose, we provided attractive patches of nesting substrate (floating rafts) for terns at a site with no availability of natural nesting habitat. Consistently with our experimental design, variation in patch size promoted establishment of small (<35 pairs) and large (>100 pairs) tern colonies. Since all habitat patches were located at the same site, our study colonies were subject to the same environmental and ecological conditions. Thus, we expected that any behavioural divergence of birds between colonies of different size would be most likely attributed to the effect of colony size per se and should not be driven by background differences in predatory pressure, food availability, habitat, etc. We combined video recording and GPS-tracking to test the following predictions: 1) initiation of communal defence is more frequent in larger colonies, while individual defence is initiated more frequently by birds from smaller colonies; 2) rate of intra-specific aggression is higher in larger colonies; 3) birds from larger colonies perform longer and more distant foraging flights (because of expected lower per capita time investment in antipredatory defence).

## Materials and methods

### Study site and experimental colonies

The study took place in 2018–2019 at Jeziorsko reservoir (51°40’N, 18°40’E), central Poland. In order to allow birds to nest in aggregations of different sizes, we provided attractive habitat patches (floating rafts) of varying size at the site with no natural nesting habitat available for common terns. The rafts were of two sizes, 10 m^2^ and 40 m^2^, allowing birds to nest in small (20–35 pairs) and large (100–130 pairs) breeding aggregations, respectively (henceforth referred to as small and large colonies). Each raft was square shaped and enclosed with a 30 cm-high mesh fence to prevent chicks from leaving the raft before fledging. The fence was unlikely to affect ability of edge nesting terns to detect approaching predators (because of transparent mesh structure) and should not restrict their mobility (no pairs nested directly next to the fence). The raft surface was elevated 0.3–0.5 m above the water level to avoid nest flooding and it was covered with sand and gravel. The first raft (small one) was installed at the reservoir in spring 2011 and it was occupied by common terns immediately during the first breeding season. During the study period (2018–2019), two large and five small rafts were available and successfully occupied by terns. Each raft was saturated with nests during the peak of the breeding season, resulting in similar between-nest distances and nest densities in our study colonies (usually 2.0–2.5 nests per 1 m^2^). Because of this negligible between-colony variation, we predicted that nesting densities were highly unlikely to explain any behavioural divergence between small and large colonies, and this variable was not included in the analyses. All rafts were located in shallow (1.0–1.5 m deep) open-water area ca. 1000 m away from the shore of the reservoir and within the borders of a strict nature reserve, which minimized human disturbance. The distances between neighbouring rafts were 200–500 m and, consequently, each colony functioned as an independent unit with limited potential for direct interactions with birds from other colonies (see [30] for details on behavioural observations supporting independent functioning of the colonies). All applicable institutional and/or national guidelines for the care and use of animals were followed during the study and all experiments were conducted by permission of the Local Bioethical Commission for Experiments on Animals and Regional Environmental Protection Directorate in Łódź, Poland.

### Foraging flight characteristics

To characterize foraging flights of common terns from small and large colonies we used ALLE 60 GPS-UHF loggers (Ecotone, Sopot, Poland). The GPS-loggers used a bidirectional radio link with base stations installed in the colonies, allowing remote data download. Loggers were deployed on 20 birds (ten individuals per colony size) at the egg incubation stage (25 May– 30 June) in 2018 and 2019. Birds were captured on nests using clap-net traps. All GPS-tracked individuals (except for one) were from edge nests (i.e. located ≤ 1m from the raft edge), which precluded testing for the effect of nest location (edge vs. central) on flight characteristics. Loggers were attached to the back feathers along the sagittal plane using two crosswise-applied strips of tape (Tesa Tape Inc., Charlotte, NC, USA). However, five individuals mechanically removed the tags, resulting in the final sample size of 15 individuals (nine and six individuals from small and large colonies, respectively). Logger mass (4.5 g including attachment) did not exceed 4% of bird body mass (in compliance with 5% threshold recommended for devices borne by flying animals [31]). Each logger recorded time and position in 5 min intervals and on average 196.1 ± 64.7 [SE] positions per individual were recorded outside the colony. Based on the geographical positions recorded by the GPS-loggers, we analysed the following foraging flights characteristics: (i) flight duration—time interval (min) between departure and return to the colony; (ii) total flight distance—the sum of the distances (km) between all positions recorded during the flight; (ii) maximum flight range–straight-line distance (km) from the colony to the most distal position recorded during the flight. In general, tagged birds mostly foraged in the shallow part of the reservoir (water depth <1.5 m; ca. 15 km^2^ area) and avoided deeper (and more distant) areas. In total, we characterized 268 individual flights. We excluded flights shorter than 15 min and those with maximal flight range <500 m from the breeding colony, as these were likely to constitute local non-foraging trips. We also excluded two extremely long flights (over two hours), as they were likely to include long resting periods outside the colony. After this preliminary data treatment, data from 209 flights were left for the analyses (mean ± SE: 13.9 ± 2.2 flights per individual).

### Defence initiation rates and aggression

We used Bushnell NatureView CamHD 119750 (Bushnell Outdoor Products, Overland Park, Kansas, USA) and InterNec i7-C54640D-IRWA (Nekma Alarm System, Łódź, Poland) audio-visual cameras to assess the rate of three types of behaviour: (i) communal defence initiation, (ii) individual defence initiation, and (iii) intra-specific aggression. In general, communal and individual defence initiation was registered with similar behavioural displays of adult terns, including an upward fight with and intense alarm vocalization. These upflights may be performed by single or few birds (individual defence initiation) or by the majority of birds from the colony (communal defence initiation). Although these kinds of defence behaviours were regularly observed in response to approaching predators in our study colonies (RW, pers. observ.), we acknowledge that some cases of upflights could also reflect false alarms, which are not rare in the common tern [32]. It is also possible that individual upflights could reflect a predatory escape, but we have never observed this kind of behaviour in adults breeding within the study colonies in response to diurnal avian predators (mostly marsh harriers *Circus aeroginosus*, white-tailed eagles *Haliaeetus albicilla*, and Caspian gulls *Larus cachinnans* at our study site), so the frequency of escape events is expected to be negligible during daylight. We cannot, however, exclude that escape behaviours are more common in response towards nocturnal ground predators, such as canids (e.g. red foxes *Vulpes vulpes* and racoon dogs *Nyctereutes procyonoides*) or mustelids (e.g. the American mink *Neovison vison*), but nocturnal predation (and tern behaviour) was not recorded in our study. Finally, upflights could also be related to other behaviours (e.g. leaving a nest to defecate or to wet the body when over-heated [32, 33]), but these should not be associated with alarm vocalization. Taking all this into account, we are convinced that most of behaviours that we recognized as defence initiation reflected response of birds to the perceived or actual danger. In fact, measurement of upflight frequencies (often with field cameras) is a standard method used to estimate the level of disturbance in the common tern colonies [34].

**Fig 1:**
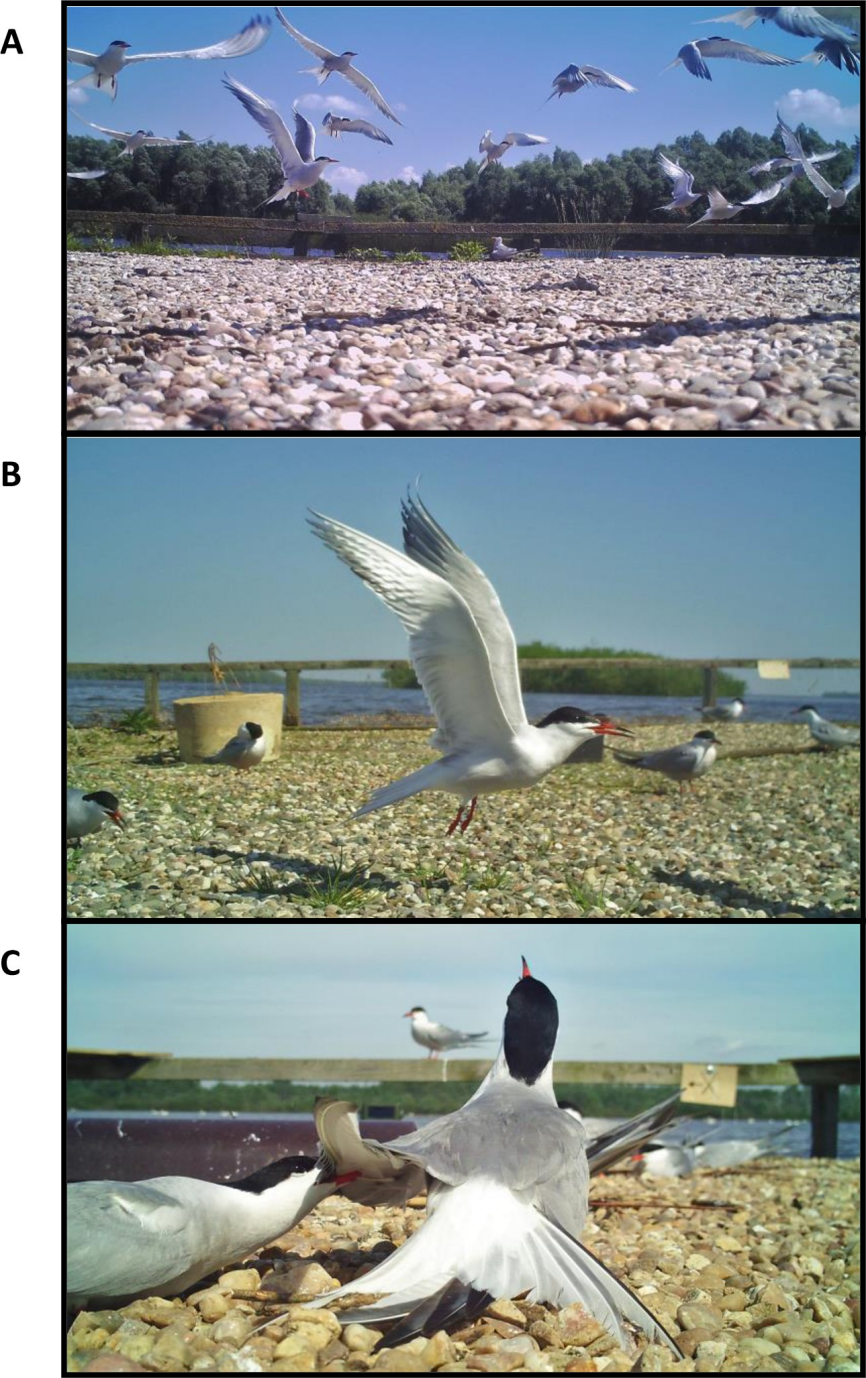
Behavioural traits assessed with field cameras: Communal defence initiation (A), individual defence initiation (B), and intra-specific aggression.

To assess communal defence initiation rate we set the cameras to record the entire colony in one frame (recording non-stop during daylight). Communal defence initiation was defined as a situation when more than half of birds from a colony took-off simultaneously following alarm calls in response to an unidentified external stimulus (e.g. predator) (Fig 1A). In all recorded cases, most birds returned to their nests within a minute from the moment of communal defence initiation. In total, we collected 289 hours of recordings for the analysis (132 and 157 hours from small and large colonies, respectively). Communal defence initiation rate was assessed both in 2018 (chick rearing stage) and 2019 (egg incubation stage). All data were collected between 05 May and 30 June.

Individual defence initiation and intra-specific aggression rates were assessed with cameras set individually to record a randomly selected nest together with its direct surrounding (3–5 neighbouring nests). All recordings were conducted at the egg incubation stage or shortly after hatching (chick age of 1–5 days), when one of the parents is continuously present at the nest. Individual defence initiation was defined as a situation when a parent made alarm calls and took-off independently from the neighbours (leaving the brood unattended) in response to an unidentified external stimulus (Fig 1B). Intra-specific aggression was defined as any aggressive interaction of a parent with a conspecific that resulted in physical contact between them (e.g. pecking, pulling a wing, brief strikes with legs or bill; Fig 1C). For the purpose of this analysis we monitored ten pairs per colony size, but data from one pair per colony size could not be reliably analysed due to low quality of recordings and, thus, discarded. Proportion of edge nests (i.e. located ≤ 1m from the raft edge) was higher in smaller colonies (70% vs. 30%) and, thus, we also tested for the effect of nest location (edge vs. centre) on both individual defence initiation and intra-specific aggression rates. In total, we analysed 624 hours of recordings (276 and 348 hours in small and large colonies, respectively) and each nest was monitored for an average of 34.7 ± 1.0 [SE] hours, only during daylight. The data were collected in 2018 between 12 and 30 June. The rate of each type of behaviour was expressed as the number of events per hour.

### Statistical analyses

Data from GPS-tracking were analysed with general linear mixed models (GLMMs), where each component of flight performance (flight duration, flight distance, and maximum flight range) was entered as a response variable in a separate model. In each model we entered colony size (small vs. large) as a fixed factor, while date and hour were entered as covariates. To test for non-linear patterns of diurnal variation in flight performance we also entered squared hour as an additional covariate, but this effect was highly non-significant in each model (all P > 0.25) and was excluded from the modelling. These linear models had similar fit to the general additive mixed models (GAMMs) (all ΔAIC < 1, as assessed with *mgcv* R package [35]), where diurnal variation was modelled using non-linear smooth functions and, thus, we reported the results of simpler GLMM models. Bird identity and year were included as random factors in each model to account for pseudoreplication resulting from multiple sampling of the same individuals and to control for inter-annual variation in behaviour.

Because collected behavioural data (communal defence initiation rate, individual defence initiation rate, and intra-specific aggression rate) showed an excess of zero values, we used generalized linear models with zero-inflated Poisson distribution for the analysis. Each variable was analysed with a separate model and included as a response variable. Colony size (small vs. large category) and breeding stage (egg incubation vs. chick rearing) were entered as fixed factors, while date, hour and squared hour were entered as covariates (to allow for non-linear diurnal variation). To test whether any possible behavioural differences between the colonies could be due to a higher proportion of edge nests at the smaller rafts we also added a fixed effect of nest location (edge vs. centre) in the analyses and presented results of both models (with and without nest location effect). In the individual models, nest identity was entered as a random factor to avoid pseudoreplication. All models were run in *glmmADMB* [36] package developed for R statistical environment [37]. Means ± SE are reported.

## Results

**Fig 2:**
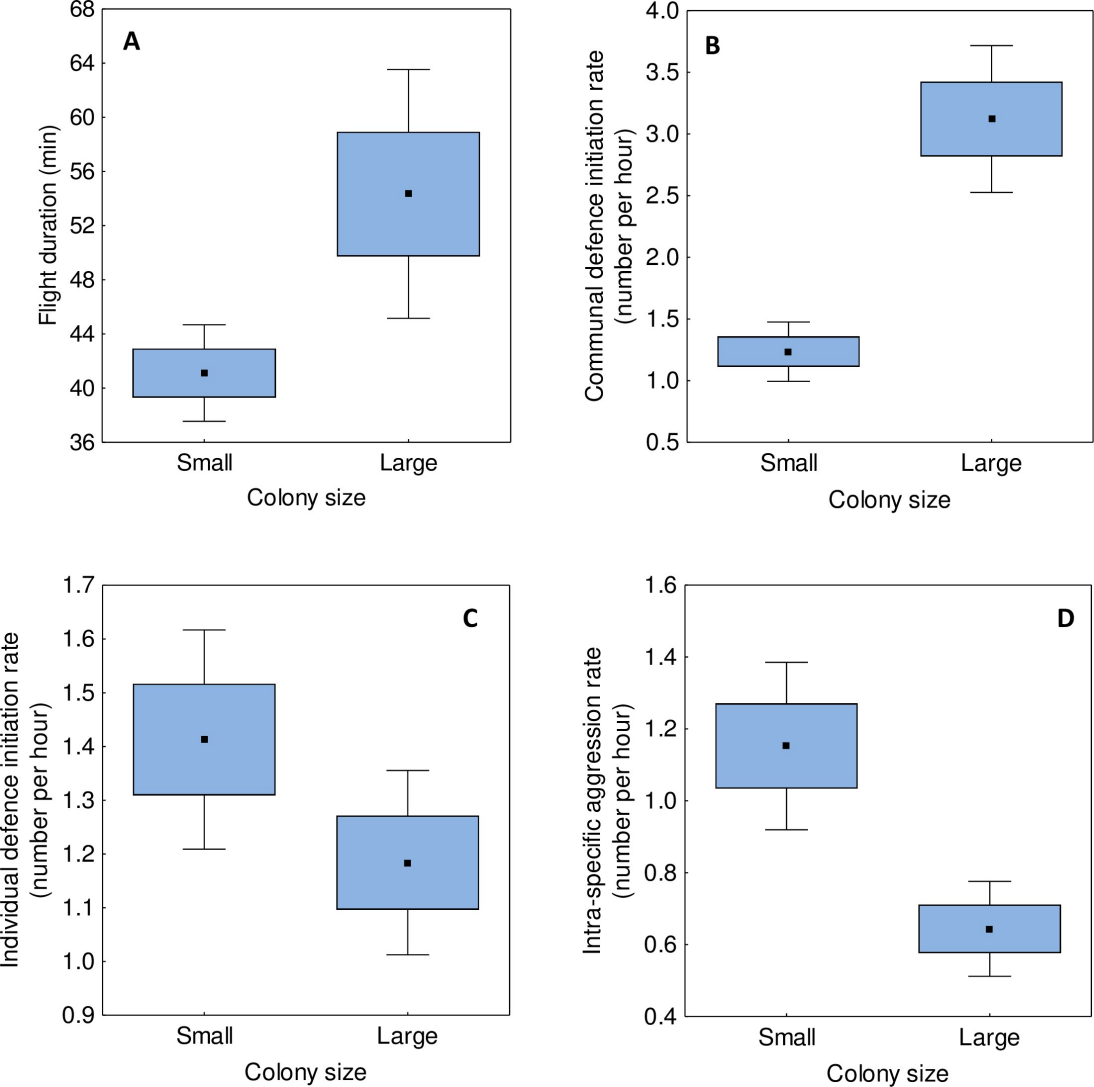
Differences in flight duration (A), communal (B) and individual (C) defence initiation rate, and intra-specific aggression rate (D) of common terns nesting in small and large colonies. Central point–mean, box–SE, whiskers– 95% confidence interval.

We found significant differences in the flight duration of terns breeding in the colonies of different size (P = 0.029, Table 1), where birds from large breeding aggregations on average spent more time outside the colony (54.3 ± 4.59 min) than birds from small aggregations (41.1 ± 1.8 min) (Fig 2A). In contrast, we found no evidence for significant differences in total flight distance (6.44 ± 0.90 km vs. 4.52 ± 0.30 km for large and small colonies, respectively) or maximum flight range between the colonies of different size (2.60 ± 0.32 km vs. 1.91 ± 0.13 km for large and small colonies, respectively) (Table 1). There was a significant diurnal variation in flight duration, as the time spent outside the colony decreased over the course of the day (β = -1.05 ± 0.35, P = 0.003, Table 1). No evidence for diurnal variation was found for total flight distance and maximum flight range (Table 1). The effect of date was found to be a non-significant predictor in all the models (Table 1).

**Table 1:** The results of general linear mixed models (Gaussian distribution) assessing variation in flight duration, total flight distance, and maximum flight range of GPS-tracked common terns nesting in small and large colonies. Bird identity and year were entered as random factors in each model.

Predictor	Flight duration			Total flight distance			Maximum flight range		
	β ± SE	z	P	β ± SE	z	P	β ± SE	z	P
Intercept	29.04 ± 56.14	0.52	0.61	**31.24 ± 13.68**	**2.28**	**0.022**	**12.06 ± 5.94**	**2.03**	**0.042**
Colony size (small vs. large)	**-15.03 ± 6.89**	**2.18**	**0.029**	-2.77 ± 1.79	1.55	0.12	-1.03 ± 0.76	1.35	0.18
Date	0.23 ± 0.36	0.62	0.52	-0.14 ± 0.09	1.63	0.10	-0.06 ± 0.04	1.42	0.16
Hour	**-1.05 ± 0.35**	**2.97**	**0.003**	-0.09 ± 0.06	1.51	0.13	-0.03 ± 0.02	1.35	0.18

Significant predictors are bolded.

The communal defence initiation rate in large colonies was significantly higher than in smaller ones (P < 0.001; Table 2; Fig 2B). A contrasting pattern was found for individual defence initiation rate, which was significantly higher in small than large colonies (P = 0.012; Table 3; Fig 2C). There was no evidence for an effect of nest location (edge vs. centre) on individual defence initiation rate (P = 0.17; Table 3). Between-colony differences were also found for intra-specific aggression rate, as terns more frequently engaged in aggressive interactions with conspecifics while nesting in small aggregations (P < 0.001; Table 3; Fig 2D). This pattern was, however, attributed to a higher proportion of edge nests in smaller breeding aggregations, as the edge breeding pairs were significantly more aggressive than the central pairs (P = 0.035; Table 3) and the effect of colony size lost significance after controlling for nest location (P = 0.13; Table 3). All types of behaviour (communal defence initiation rate, individual defence initiation rate and intra-specific aggression rate) were more frequent at chick stage than during egg incubation (all P < 0.001; Tables 2 and 3). Also, all behaviours showed non-linear diurnal variation, although the patterns varied between traits. Communal defence initiation rate was lowest during the midday hours (positive quadratic trend; Table 1), while the opposite was found for individual defence initiation and aggression rates (negative quadratic trend, marginally non-significant for individual flight initiation rate; Tables 2 and 3).

**Table 2:** The results of generalized linear model (zero-inflated Poisson distribution) assessing variation in communal defence initiation rate of common terns nesting in small and large colonies.

Predictor	Communal defence initiation		
	β ± SE	z	P
Intercept	4.10 ± 2.16	1.90	0.058
Colony size (small vs. large)	**-0.98 ± 0.17**	**5.79**	**<0.001**
Breeding stage (chicks vs. eggs)	**1.62 ± 0.51**	**3.18**	**0.001**
Date	-0.012 ± 0.012	0.99	0.32
Hour	**-0.20 ± 0.04**	**5.33**	**<0.001**
Hour squared	**0.010 ± 0.001**	**6.85**	**<0.001**

Significant predictors are bolded.

**Table 3:** The results of generalized linear mixed models (zero-inflated Poisson distribution) assessing variation in individual defence initiation rate and intra-specific aggression rate of common terns nesting in small and large colonies. Models with and without nest location (edge vs. centre) are presented. Nest identity was entered as a random factor in each model.

Predictor	Individual defence initiation			Intra-specific aggression		
	β ± SE	z	P	β ± SE	z	P
Intercept	**-13.78 ± 4.36**	**3.16**	**0.002**	**-13.07 ± 5.66**	**2.31**	**0.021**
Colony size (small vs. large)	**1.06 ± 0.42**	**2.51**	**0.012**	**1.20 ± 0.57**	**2.1**	**0.035**
Breeding stage (chicks vs. eggs)	**0.36 ± 0.15**	**2.39**	**0.017**	**0.71 ± 0.19**	**3.75**	**<0.001**
Date	**0.08 ± 0.02**	**3.17**	**0.002**	**0.07 ± 0.03**	**2.08**	**0.037**
Hour	0.04 ± 0.03	1.11	0.27	**0.13 ± 0.05**	**2.78**	**0.006**
Hour squared	**-0.003 ± 0.001**	**2.11**	**0.035**	**-0.005 ± 0.002**	**2.81**	**0.005**
Intercept	**-13.94 ± 4.35**	**3.20**	**0.001**	**-13.34 ± 5.64**	**2.37**	**0.018**
Colony size (small vs. large)	**0.88 ± 0.43**	**2.04**	**0.041**	0.85 ± .057	1.5	0.13
Nest location (edge vs. centre)	0.41 ± 0.30	1.38	0.17	**0.82 ± 0.39**	**2.11**	**0.035**
Breeding stage (chicks vs. eggs)	**0.36 ± 0.15**	**2.43**	**0.015**	**0.71 ± 0.19**	**3.82**	**<0.001**
Date	**0.08 ± 0.02**	**3.19**	**0.001**	**0.07 ± 0.03**	**2.09**	**0.037**
Hour	0.04 ± 0.03	1.12	0.26	**0.13 ± 0.05**	**2.78**	**0.006**
Hour squared	**-0.003 ± 0.001**	**2.11**	**0.035**	**-0.005 ± 0.002**	**2.81**	**0.005**

Significant predictors are marked in bold.

## Discussion

The results of our study provide strong evidence for a spectrum of behavioural differences associated with colony size in the common tern. Most importantly, we found that birds from larger colonies more frequently engaged in communal defence and they performed longer foraging flights, while terns from smaller colonies more frequently showed individual defence behaviours. Also, inconsistently with our prediction, we found that intra-specific aggression rate was higher in smaller colonies, but this pattern was primarily due to a higher proportion of edge breeding pairs, which were more aggressive. Because of our experimental design, in which we promoted establishment of large and small tern colonies under uniform environmental and ecological conditions, we suggest that observed variation in certain behavioural traits (defence rates and flight duration) can be directly attributed to differences in the size of reproductive groups.

Communal defence is recognized as an important advantage of colonial breeding in birds and its efficiency is expected to increase with group size [15]. Here, we found that the type of defence behaviour was associated with colony size in the common tern, as birds from larger colonies were more likely to engage in communal defence. In contrast, terns from smaller colonies more frequently initiated defence activities on individual basis. These differences can be attributed to several non-exclusive mechanisms. First, the benefit/cost ratio of communal defence correlates with the number of mobbers [15], however, this relationship is likely to be of non-linear character and there may be a certain group size threshold, below which communal defence is ineffective and does not allow to successfully deter predators. Under such circumstances, the risk of death or injury during mobbing may override negligible benefits of unsuccessful communal defence, and some (or majority of) individuals may be reluctant to engage in this type of behaviours. Irrespectively of the cost/benefit ratios, individuals could possibly sort between colonies by their general responsiveness to social cues. Boldest individuals are most likely to individually initiate defence activities against predators and it should largely depend on the social susceptibility of shier conspecifics whether they join in the communal defence or not. Thus, assuming that larger social groups attract more socially responsive individuals, we should expect a higher rate of communal, but not individual, defence rate in larger colonies, consistently with our results. However, we lack any empirical data to test this hypothesis and it remains to be determined in the future research, whether behavioural divergence (in terms of communal defence rates) of birds between colonies of different sizes constitutes a response to the colony size per se, or whether it rather reflects divergence in the personalities of individuals attracted to the colonies of different size.

We also found that terns from larger colonies performed longer foraging flights, although differences in the total flight distance and maximum flight range between birds from large and small colonies were non-significant. This suggests that there is little differentiation in the location of foraging grounds among the colonies, but individuals from larger colonies could afford to spend more time foraging. This pattern could possibly reflect a lower per capita time investment in brood defence by birds breeding in the larger aggregations. The most solid empirical evidence for a negative correlations between individual time investment in defence-associated behaviours (mostly vigilance) and group size comes from mammals [e.g. 38–40]. In birds, similar associations have been reported for several taxa. For example, the rate of individual vigilance bouts decreased with group size (ranging from one to twelve individuals) in the greater rheas *Rhea americana* during the non-breeding period, although an average length of each bout remained relatively constant [41]. Also, the percentage of cliff swallows *Hirundo pyrrhonota* that were alert declined with flock size and individuals in large flocks spent less time alert and had more time to engage in other activities such as preening and sun-bathing [16]. A comprehensive meta-analysis of over 150 avian behavioural studies indicated weak to moderate negative correlations between group size and time spent vigilant, scan frequency, or scan duration [42]. In contrast, associations of foraging flight characteristics with colony size have been analysed mostly with respect to intra-specific competition for food. It is widely recognized that foraging range and duration of foraging trips of central-place foragers increase with colony size due to prey depletion or disturbance (so called Ashmole’s halo [43–45]). However, empirical evidence for this pattern is valid for spatially dispersed colonies, where home ranges of adjacent colonies do not overlap or the degree of an overlap is small [45]. Our study colonies were located at the same site and at short distances from each other (200–500 m distance for neighbouring colonies), so birds from colonies of different size had to use the same foraging areas. Thus, under our experimental design, individuals from small and large colonies competed for the same food resources (mostly common bleak *Alburnus alburnus* and roach *Rutilus rutilus*, as indicated by inspection of food items found in the colonies) and food depletion should have similar impact on their foraging flights. Although we have no data on changes in prey densities at our study site, we suggest that longer flight duration of terns from larger colonies is unlikely to be explained with higher intra-specific competition for food, but this pattern is likely to be specific for our experimental design and it does not contradict empirical evidence for the occurrence of Ashmole’s halos around avian colonies.

Finally, contrary to our expectation, we found that the rate of intra-specific aggression was higher in smaller colonies. We predicted that the frequency of agonistic interactions should increase with the number of adult individuals present in the colony and that higher aggression rate in larger aggregations could be facilitated by appropriate hormonal up-regulation in birds subject to elevated intra-specific competition. For example, testosterone-implanted yellow-legged gull *Larus cachinnans* males were more aggressive and acquired larger territories under high nest densities, where strong competition for space and mates occurred [20]. Although competition for nesting sites is indeed likely to be higher in our large experimental colonies, we found that this was accompanied with lower intra-specific aggression of terns at the incubation and early post-hatching stages. This pattern could likely be explained with a lower proportion of edge breeding pairs in the larger colonies. The edge pairs are usually at more risk than the central pairs, as they and their broods may be more easily accessed by predators [46]. Thus, individuals nesting at the colony edges may show a greater stress-mediated reactivity to external stimuli and they may have adaptatively elevated testosterone levels, as high concentration of testosterone should increase investment in nest defence against predators [47]. At the same time, elevated testosterone levels are expected to increase aggression rate against conspecifics, which would be consistent with our behavioural observations. The differences between edge and centre nest locations could be even more pronounced in breeding aggregations larger than our experimental colonies. In natural conditions, common terns can nest in colonies of up to several thousand pairs [48] and our experimental colonies (both small and large) were at the lower end of the natural range of colony sizes in this species. However, since we demonstrated behavioural divergence of terns breeding across a limited spectrum of colony sizes, we predict that differences in behaviour should be even more apparent under a greater variation in group size, which could possibly be tested under natural non-experimental conditions.

In conclusion, our study provided evidence for marked behavioural divergence of common terns breeding in aggregations of different size. The results suggest that different colony sizes may be associated with different foraging strategies and behavioural syndromes comprising diverse personality traits such as boldness towards predators and social responsiveness. It remains to be tested whether these behavioural differences reflect processes of phenotypic sorting or whether they are a result of behavioural plasticity under different social contexts.

## Supporting information

S1 Data (XLSX)
